# Plasma levels of S100B and neurofilament light chain protein in stress-related mental disorders

**DOI:** 10.1038/s41598-022-12287-1

**Published:** 2022-05-18

**Authors:** Johanna Wallensten, Fariborz Mobarrez, Marie Åsberg, Kristian Borg, Aniella Beser, Alexander Wilczek, Anna Nager

**Affiliations:** 1 Academic Primary Health Care Centre, Region Stockholm, Solnavägen 1E, Box 45436, 104 31 Stockholm, Sweden; 2 Department of Clinical Sciences, Karolinska Institutet, Danderyd University Hospital, 18288 Stockholm, Sweden; 3 Department of Medical Sciences, Uppsala University, 75185 Uppsala, Sweden; 4 Division of Family Medicine and Primary Health Care, Department of Neurobiology, Care Sciences and Society, Karolinska Institutet, 17177 Stockholm, Sweden

**Keywords:** Biomarkers, Medical research, Pathogenesis

## Abstract

The pathophysiological changes underlying stress-related mental disorders remain unclear. However, research suggests that alterations in astrocytes and neurons may be involved. This study examined potential peripheral markers of such alterations, including S100B and neurofilament light chain (NF-L). We compared plasma levels of S100B and NF-L in patients with chronic stress-induced exhaustion disorder (SED), patients with major depressive disorder (MDD), and healthy controls. We also investigated whether levels of S100B and NF-L correlated with levels of astrocyte-derived extracellular vesicles (EVs that indicate astrocyte activation or apoptosis) and with symptom severity. Only women had measurable levels of S100B. Women with SED had higher plasma levels of S100B than women with MDD (*P* < 0.001) and healthy controls (*P* < 0.001). Self-rated symptoms of cognitive failures were positively correlated with levels of S100B (r_s_ = 0.434, *P* = 0.005) as were depressive symptoms (r_s_ = 0.319, *P* < 0.001). Plasma levels of astrocyte-derived EVs were correlated with levels of S100B (r_s_ = 0.464, *P* < 0.001). Plasma levels of NF-L did not differ between the groups and were not correlated with symptom severity or EV levels. Thus, long-term stress without sufficient recovery and SED may be associated with raised plasma levels of S100B, which may be evidence of pathophysiological changes in astrocytes. The findings also support the hypothesis that plasma levels of S100B are associated with cognitive dysfunction.

## Introduction

Chronic stress increases the risk for psychiatric disorders such as major depressive disorder^1^ (MDD) and chronic stress-induced exhaustion disorder (SED), also called clinical burnout. SED is caused by long-term stress without sufficient recovery, which leads to mental and physical exhaustion^2,3^. Stress impacts cognitive processes^4^, and cognitive symptoms such as impaired memory and attention are common in patients with SED^3^. SED is included in the Swedish version of the International Classification of Diseases, tenth version (ICD-10)^5^ (Table 1).

**Table 1:** Diagnostic criteria for stress-induced exhaustion disorder (SED) according to the Swedish National Board of Health and Welfare.

A. Physical and mental symptoms of exhaustion for at least 2 weeks. The symptoms have developed in response to one or more identifiable stressors present for at least 6 months
B. The clinical picture is dominated by markedly reduced mental energy, as manifested by reduced initiative, lack of endurance, or increased time needed for recovery after mental effort
C. At least four of the following symptoms have been present, nearly every day, during the same 2-week period Concentration difficulties or impaired memory Markedly reduced capacity to tolerate demands or to work under time pressure Emotional instability or irritability Sleep disturbance Marked fatigability or physical weakness Physical symptoms such as aches and pains, palpitations, gastrointestinal problems, vertigo, or increased sensitivity to sound
D. The symptoms cause clinically significant distress or impairment in occupational, social, or other important respects
E. The symptoms are not due to the direct physiological effects of a substance (e.g., a drug of abuse, a medication) or a physical illness/injury (e.g., hypothyroidism, diabetes, infectious disease)

Criteria A–E must be fulfilled to diagnose SED.

Chronic neuroinflammation may underlie not only MDD^6^, but also SED. This neuroinflammatory model posits that chronic inflammation affects glial cell function, including the function of astrocytes. The model is in line with research into pathophysiological changes that suggests that stress-related mental disorders are associated with alterations in astrocyte density and morphology in specific regions of the brain^7–10^. Potential markers of alterations in astrocytes include astrocyte-derived extracellular vesicles (EVs), which are released from the cell membrane during cell activation and/or apoptosis^11^. These EVs can be measured in plasma with flow cytometry by labeling them with antibodies for astrocyte-derived proteins such as glial fibrillary acidic protein (GFAP) and aquaporin 4 (AQP4). We recently demonstrated higher levels of astrocyte-derived EVs in the plasma of patients with SED and MDD than in healthy controls. Furthermore, levels of these EVs were significantly higher in patients with SED than patients with MDD, which suggests that different pathophysiological processes may underlie these stress-related mood disorders^12^.

Affected astrocytes can also be detected in peripheral blood with validated commercial assays, such as assays for S100B. S100B is a cytosolic calcium-binding protein^13^ synthesized primarily by astrocytes^14^. Raised levels of S100B are used clinically as an indicator of brain damage^15^ and may be a marker of neurological disorders^16^. S100B is hypothesized to be a marker of blood brain barrier integrity^17^ and has been found in the cerebrospinal fluid (CSF) and serum of patients with mood disorders, including MDD^18–21^. Its potential use as a marker of altered astrocytes in Alzheimer disease^22^ is additional evidence of its link with cognitive dysfunction and possibly with chronic stress and neuroinflammation. To the best of our knowledge, however, no previous studies have investigated S100B in patients with SED.

Although astrocytes are the main source of S100B, this calcium-binding protein can also be synthesized in cells outside the nervous system, such as adipocytes and melanocytes^23^, and its levels in serum can increase after trauma, such as acute fracture^24^. Thus, S100B might not always come from changes in astrocytes. It is therefore useful to investigate whether any raised levels of S100B in patients with stress-related mental disorders are correlated with increased levels of astrocyte-derived EVs in those patients.

Another marker that has been explored in people with psychiatric disorders is cytoskeletal protein neurofilament light chain (NF-L). NF-L, which is expressed in central^25^ and peripheral^26^ neurons, is released in a variety of physiological processes, such as aging^27^, and is a potential blood biomarker of neuro-axonal injury^28^. Increased levels of NF-L have been found in the CSF of people with psychiatric disorders, such as bipolar disorder^29^, and in plasma in people with treatment-resistant major depression^30^.

The primary aim of this study was to compare plasma levels of S100B and NF-L in patients with SED, patients with MDD, and healthy controls. The secondary aim was to investigate whether these levels correlated with levels of astrocyte-derived EVs and with symptom severity in these three groups.

## Methods

### Study design and participants

From 2014 to 2018, patients at a psychiatric outpatient clinic in Stockholm who fulfilled the diagnostic criteria for SED^5^ (Table 1) or MDD^31^ were consecutively invited to participate in the study. Patients were included if they were between the ages of 18 and 65 years, could speak Swedish, and had no somatic or psychiatric disorders other than SED or MDD (including substance use disorders). Blood samples were used to exclude patients with thyroid disease, anemia, vitamin B12 deficiency, and alcohol overconsumption. Patients were excluded if they had a history of head trauma with loss of consciousness. A physician conducted a clinical examination. The Swedish version of the Mini International Neuropsychiatric Interview (M.I.N.I.) 6.0.0^32,33^ was used to diagnose mental disorders other than SED. M.I.N.I. was administered by a clinic physician, one of the researchers, or a psychologist familiar with the instrument. Patients with SED who also fulfilled the criteria for MDD were included in the SED group if the physician judged that the MDD was secondary to the SED (n = 2). A total of 31 patients with SED and 13 with MDD were recruited from this clinic. To increase the number of patients with MDD in the study, patients at an additional psychiatric outpatient clinic who fulfilled the ICD-10 criteria for MDD were also invited to participate, resulting in a total of 31 patients with MDD in the study (Supplemental Fig. 1). To minimize the possibility that patients with SED had experienced stress symptoms for different lengths of time prior to blood sampling, patients were only included if they had been diagnosed less than three months before the start of the study.

The healthy controls in the study came from a group recruited in 2009 to serve as controls in future studies. A total of 1146 adult permanent residents of Sweden age 55 and below, were randomly selected by Statistics Sweden, Sweden’s official government statistics agency. Those who agreed to participate were contacted by phone for a first screening. If they described themselves as healthy, they were invited to a medical investigation that included a clinical and a psychological examination performed by a physician. People with current or previous diagnoses of SED or other mental disorders, cancer, endocrine disorders, or cardiovascular disorders were excluded, resulting in 165 healthy controls. The current study included 61 of these healthy controls, matched for sex and age with patients with SED or MDD.

The regional Ethical Review Board in Stockholm, Sweden (http://www.epn.se/en/start/), approved the study (Dnr. 2008/0061-31, 2014/585-31/1, 2016/1239-32, 2017/2088-32, 2021-00346 and 2021-05515-02). The study was conducted in keeping with the principles outlined in the Declaration of Helsinki. The researchers provided potential participants with oral and written information about the study and obtained written informed consent prior to inclusion.

Prior to linkage and analysis, data were pseudonymized.

### Symptom rating scales

To quantify the severity of depressive symptoms, we used the self-assessment version of the Montgomery Åsberg Depression Rating Scale (MADRS-S)^34^. MADRS-S includes nine items about sadness, inner tension, sleep, appetite, concentration, lassitude, ability to feel, pessimistic thoughts, and suicidal thoughts. Overall scores range from 0 to 54 points. To measure the experience of cognitive failures in daily life, we used the 25-item Cognitive Failures Questionnaire (CFQ)^35^. The CFQ includes 25 questions about failures of perception, memory, and motor function. Overall scores range from 0 to 100 points. All three groups (patients with SED, patients with MDD, and healthy controls) completed MADRS-S. The CFQ was completed by patients with SED and patients with MDD but not healthy controls.

### Sample collection

Blood samples were drawn at inclusion from an antecubital vein after at least 15 min of rest. They were drawn in the morning, and the patients were asked to fast for 12 h prior to sampling. They were also asked to avoid physical activity prior to blood sampling. The samples were drawn into citrated tubes and centrifuged within 1 h at room temperature for 20 min at 2000×*g*. After centrifugation, the platelet poor plasma was stored at − 80 °C. The blood sampling routines for the healthy controls were similar to the routines for the patients, and the samples from all three groups were analyzed in the same batch. Because the samples from controls were collected in 2009, these samples had been stored at least 5 years longer than the samples from the patients at the time of analysis.

### Analysis of S100B and neurofilament light chain protein

A commercially available enzyme-linked immunosorbent assay (ELISA) was used to measure S100B (Abcam, Cambridge, United Kingdom) and NF-L (Nordicbiosite, Stockholm, Sweden) in plasma. The analyses were conducted in accordance with the manufacturer’s protocols. The analyses of S100B could not detect levels < 0.011 ng/ml in our samples.

### Analysis of astrocyte-derived extracellular vesicles

We used flow cytometry to measure astrocyte-derived EVs. The method is described in greater detail in a previous publication^12^. Briefly, platelet poor plasma samples were thawed and centrifuged at 2000×*g* for 20 min at room temperature **(**RT). The upper supernatant was then high-speed centrifuged for 45 min at RT (at 20,800×*g*) to obtain an EV-enriched pellet. To analyze astrocyte-derived EVs, 20 µl of the pellet were labeled with anti-Aquaporin-4 Dylight 488 (Abcam, Cambridge, UK) and anti-GFAP Dylight 755 (Abcam, Cambridge, UK) and incubated in darkness for 20 min. The EVs were measured on a Beckman Gallios instrument (Beckman coulter, Brea, CA, USA). The EVs were defined by size (forward scatter) (~ 0.3–0.9 µm in diameter) and by co-expression of AQP4 and GFAP.

### Statistical methods

To compare clinical and demographic characteristics, we used unpaired t-tests or the Mann–Whitney test to compare two groups and the Kruskal–Wallis test with the Bonferroni correction to compare three groups. The Fisher’s exact test was used to compare antidepressant medication and selective serotonin reuptake inhibitors or serotonin and norepinephrine reuptake inhibitors (SSRIs or SNRIs) in patients with SED and patients with MDD. The Kruskal–Wallis test with the Bonferroni correction was used to compare levels of S100B and NF-L in the three groups (patients with SED, patients with MDD, and healthy controls).

Because of the non-normal distribution of the data, the two-way Spearman’s correlation test was used to analyze correlations between plasma levels of two variables, S100B and NF-L, and astrocyte-derived EVs, symptom severity scale scores and age. The results are presented as Spearman coefficients (r_s_). The Mann–Whitney test was used to compare plasma levels of S100B and NF-L in women and men.

For all statistical tests, *P* < 0.05 was considered statistically significant. We did not adjust for multiple testing.

## Results

### Clinical and demographic characteristics

A total of 31 patients with SED, 31 patients with MDD, and 61 healthy controls were included in the study. The groups did not differ significantly by age, sex, or BMI (Table 2). Self-reported severity of depressive symptoms (MADRS-S) reflected clinical expectations. That is, patients with MDD had the highest level of depressive symptoms, significantly higher than healthy controls (*P* < 0.001), but not significantly higher than patients with SED (*P* = 0.407) (Table 2). Self-reported severity of cognitive symptoms (CFQ) also reflected clinical expectations: patients with SED reported significantly more severe cognitive symptoms than patients with MDD (*P* = 0.025).

**Table 2:** Clinical and demographic characteristics of patients with stress-induced exhaustion disorder (n = 31), patients with major depressive disorder (n = 31), and healthy controls (n = 61).

Clinical and demographic characteristics	Stress-induced exhaustion disorder	Major depressive disorder	Healthy controls	*P* value
Mean age in years	n = 31 44.6 (9.7)	n = 31 40.3 (10·8)	n = 61 42.2 (9.5)	0.206
Women	n = 31 27 (87.1%)	n = 31 26 (83.9%)	n = 61 52 (85.2%)	0.999
Mean BMI	n = 31 24.8 (5.5)	n = 31 25.0 (5.0)	n = 61 24.7 (3.6)	0.898
Antidepressant medication	n = 30 25 (83.3%)	n = 29 24 (82.8%)	n = 0	1.000^a^
SSRI or SNRI	n = 25 25 (100%)	n = 24 21 (87.5%)	n = 0	0.110^a^
Mean CFQ score	n = 14 57.7 (11.0)	n = 26 50.0 (12.1)	n = 0	0.025*^a^
Mean MADRS-S score	n = 22 19.9 (5.6)	n = 25 27.1 (7.9)	n = 61 5.0 (3.6)	< 0.001***^b^

Data are mean (SD) or n (%).

*BMI* body mass index, *CFQ* cognitive failures questionnaire, *MADRS-S* self-reported version of the Montgomery–Åsberg Depression Rating Scale, *SNRI* serotonin and norepinephrine reuptake inhibitor, *SSRI* selective serotonin reuptake inhibitor.

^a^The *P* value represents the difference between patients with stress-induced exhaustion disorder and patients with major depressive disorder.

^b^The *P* value represents the results of the comparison of all three groups. Further analyses showed a statistically significant difference between patients with stress-induced exhaustion disorder and healthy controls and between patients with major depressive disorder and healthy controls.

### Comparison of levels of S100B and neurofilament light chain protein

**Figure 1:**
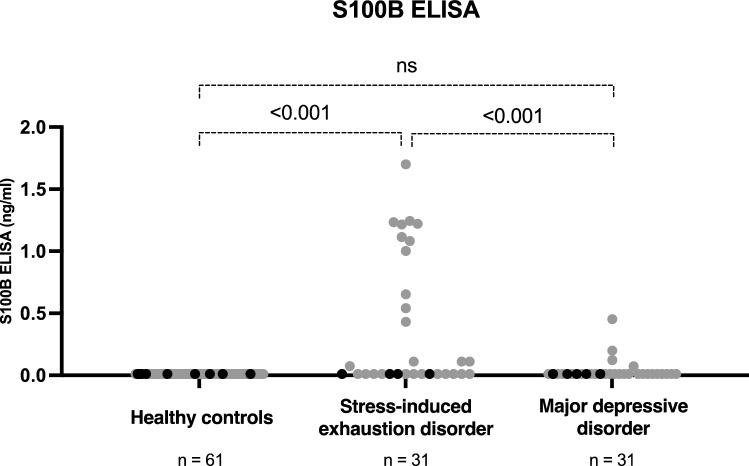
Plasma levels of S100B measured by enzyme-linked immunosorbent assay (ELISA). Gray dots represent women and black dots represent men.

Plasma levels of S100B were lower than the ELISA assay could measure (< 0.011 ng/ml) in 61 healthy controls (100% of healthy controls), 27 of 31 patients with MDD (87% of patients with MDD), and 17 of 31 patients with SED (55% of patients with SED). Only women had measurable levels of S100B (14 of 27 women with SED and 4 of 26 with MDD). The non-parametric tests showed that patients with SED had significantly higher plasma levels of S100B than patients with MDD (*P* < 0.001) and healthy controls (*P* < 0.001), but there was no significant difference between plasma levels of S100B in patients with MDD and healthy controls (*P* = 0.406) (Fig. 1). In patients with SED, mean levels of S100B were 0.388 ng/ml, in patients with MDD, 0.037 ng/ml, and in healthy controls, 0.011 ng/ml.

Levels of NF-L did not differ significantly in the groups (*P* = 0.104) or by sex (*P* = 0.391) (Supplementary Fig. 2).

### Symptom severity

**Figure 2:**
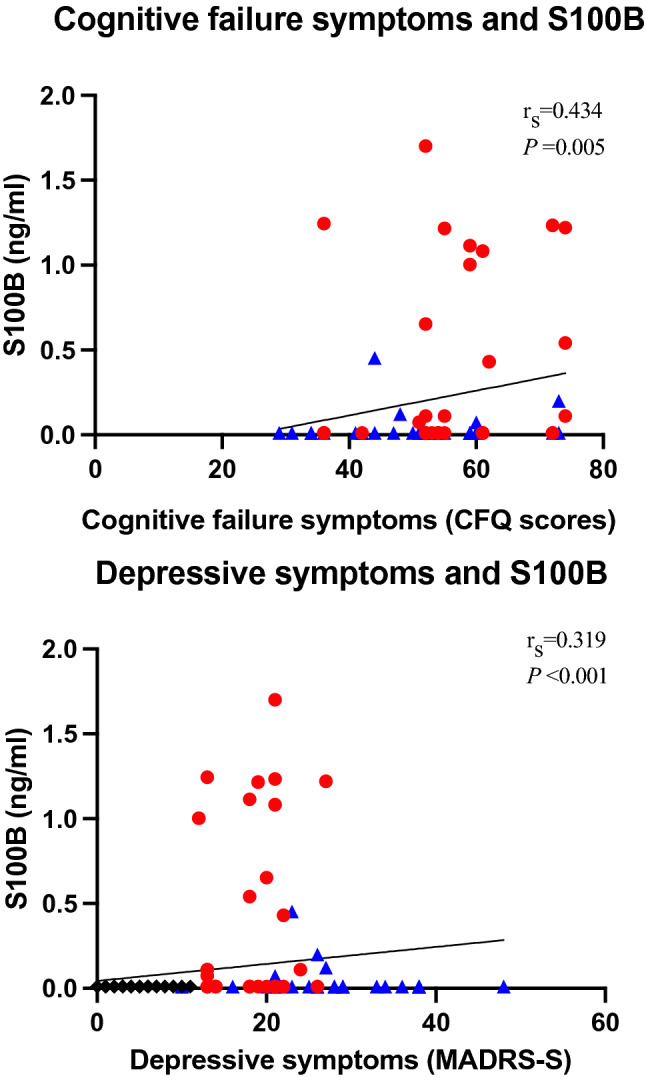
Correlation between levels of S100B and severity of self-rated symptoms of cognitive failure measured with the Cognitive Failure Questionnaire (CFQ) and severity of self-rated depressive symptoms measured with the self-assessment version of the Montgomery Åsberg Depression Rating Scale (MADRS-S)*.* Red filled circle = patients with stress-induced exhaustion disorder; Blue filled triangle = patients with major depressive disorder and black filled diamond = healthy controls.

Healthy controls did not provide information about symptoms of cognitive failure (did not complete the CFQ) and were therefore not included in the analysis. When the patient groups were combined, self-rated symptoms of cognitive failures were significantly positively correlated with levels of S100B (r_s_ = 0.434, *P* = 0.005) (Fig. 2). This finding was observed only in women; no men had measurable levels of S100B.

All groups provided information about depressive symptoms (MADRS-S), and therefore all groups were included in the analysis. Self-rated depression severity was significantly positively correlated with levels of S100B when all three groups were combined (r_s_ = 0.319, *P* < 0.001) (Fig. 2).

Neither symptoms of cognitive failure (r_s_ =  − 0.213, *P* = 0.187) nor of depression (r_s_ = 0.169, *P* = 0.080) were correlated with levels of NF-L in either men or women.

### Age

Age was not correlated with plasma levels of S100B (r_s_ = 0.050, *P* = 0.586) or NF-L (r_s_ = 0.077, P = 0.398).

### Astrocyte-derived extracellular vesicles

**Figure 3:**
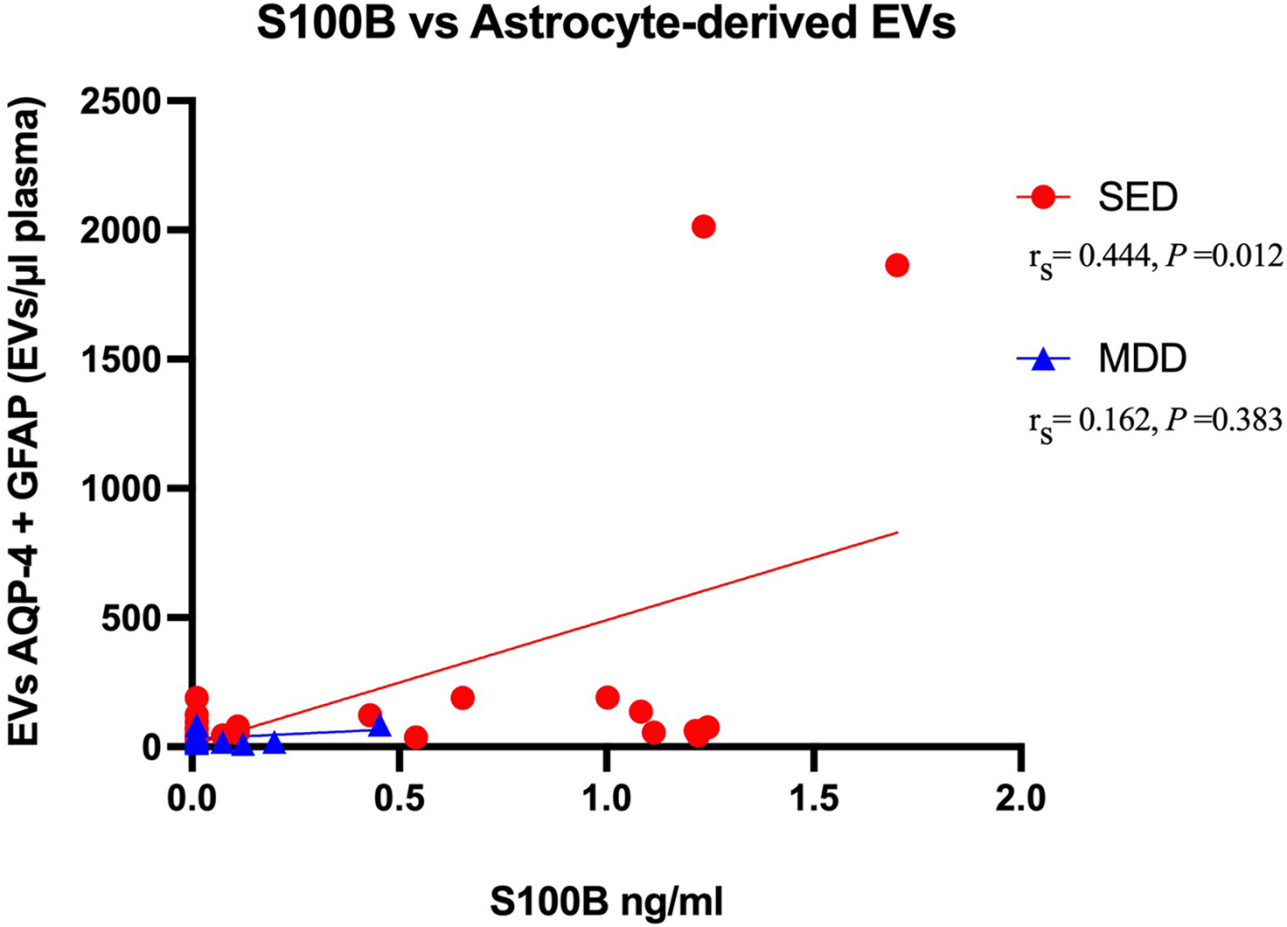
Rank correlation between plasma levels of S100B and astrocyte-derived extracellular vesicles (EVs) in patients with stress-induced exhaustion disorder and patients with major depressive disorder. Red filled circle = patients with stress-induced exhaustion disorder, blue filled triangle = patients with major depressive disorder. Healthy controls are not shown because levels of S100B were not measurable in this group.

In a previous analysis of the blood samples from this study population^12^, our group observed significantly higher plasma levels of astrocyte-derived EVs in the patients with SED and in the patients with MDD than in the healthy controls. In the current study, levels of S100B were positively correlated with levels of astrocyte-derived EVs when comparing all groups together r_s_ = 0.464, *P* < 0.001). Levels of S100B were positively correlated with levels of astrocyte-derived EVs in patients with SED (r_s_ = 0.444, *P* = 0.012) but not in patients with MDD (r_s_ =  − 0.162, *P* = 0.383). Since healthy controls did not have measurable levels of S100B correlation was not estimated (Fig. 3). No correlation was observed between levels of NF-L and levels of astrocyte-derived EVs when all groups were analyzed together (r_s_ = 0.096, *P* = 0.291).

## Discussion

In this study, we observed that patients with SED had significantly higher plasma levels of S100B than patients with MDD and healthy controls. Self-rated symptoms of cognitive failures were significantly positively correlated with levels of S100B, as were depressive symptoms. Plasma levels of astrocyte-derived EVs were significantly correlated with levels of S100B. Plasma levels of NF-L did not differ between the groups and were not correlated with symptom severity or levels of astrocyte-derived EVs.

Research into S100B and chronic stress is in its infancy. Elevated plasma levels of S100B have been observed in rats after restraint stress^36^. However, this is the first investigation into levels of S100B in patients with fatigue and cognitive symptoms due to chronic stress without adequate recovery (that is, in patients with SED). Stress is a risk factor for psychiatric disorders, including MDD^37^, and elevated levels of S100B have previously been found in serum and cerebral spinal fluid in people with mood disorders, including MDD^21,38^.

The mean level of S100B in patients with SED in the current study (0.388 ng/ml) exceeded a suggested reference level used to identify pathological CT scans in hospital emergency settings after traumatic brain injury (≥ 0.105 ng/ml)^15^. However, caution must be used in interpreting the meaning of this difference, as the previous study used a different method to measure serum levels of S100B (the Elecys^®^ S100 assay on a Cobas e411 instrument, both from Roche Diagnostics, Germany). In the present study, both the mean level of S100B in healthy controls and the mean level in patients with MDD were under the reference level used to identify traumatic brain injury in the earlier study.

We did not observe significantly higher levels of S100B in the peripheral blood of patients with MDD than in healthy controls. Other studies have observed such a difference^38^. The discrepancy in findings may be explained by the small sample size in our study and the method we used to measure S100B, which cannot detect levels of < 0.011 ng/ml. These methodological limitations may also lie behind our finding of significantly higher plasma levels of S100B in patients with SED than patients with MDD. On the other hand, patients with chronic stress without adequate recovery might experience different pathophysiological processes than patients with MDD. This discrepancy would only be apparent when the two groups are analyzed separately, as in the present study.

In our study, regardless of diagnosis, only women had measurable levels of S100B. This finding supports the results of previous research on patients with MDD that suggest that gender might affect levels of S100B^39^. Gender may also affect neurobiological cerebral vulnerability to stress. According to research on patients with SED, abnormalities such as cortical thinning and amygdala enlargement were more pronounced in women than men^40^. However, our findings should be interpreted with caution because of the small size of our study population and the limited number of patients with elevated levels of S100B.

In our study, intake of antidepressant medication did not differ significantly between patients with SED and patients with MDD. Previous research in an animal model has shown that SSRIs may prevent psychosocial stress from reducing the number of astrocytes in the hippocampus^41^. This finding is consistent with observations that antidepressant medication can reduce levels of S100B in serum^18,42^. It is thus possible that the patients in the current study would have had higher levels of S100B if they had not been taking antidepressants.

Like our study, several previous studies have found that levels of S100B are associated with cognitive outcomes^39,43–45^. In most prior studies, higher levels are linked with negative cognitive outcomes^39,44,45^, such as cognitive impairment in people with small vessel disease^45^. An exception to this pattern was a study of cognitive function in people with type 2 diabetes, which found that higher levels of S100B were associated with better cognitive function^43^.

In the present study, we observed a positive correlation between S100B and astrocyte-derived EVs that is mainly driven by the patients with SED (primarily two individuals). The clinical implication is somewhat unclear, and larger studies are needed to understand the mechanism behind these findings. However, since both S100B and EVs that are positive for both AQP4 and GFAP derive from astrocytes, astrocytes could play an important role in the disease mechanism in patients with SED. Moreover, flow cytometric assays used to measure EVs have the advantage of measuring specific populations (i.e., vesicles only) and simultaneously enable further phenotyping of vesicles, that is, the use of several antibodies at once. Based on the results presented here and previously by our group, flow cytometry also seems to be more sensitive than ELISA in analyzing soluble proteins, such as CD40L^46^ and HMGB1^47^.

### Limitations

There are several limitations in this study. The healthy controls were recruited several years before the patients. However, the blood sampling procedure was similar for patients and healthy controls, and blood samples were analyzed in the same batch. Storage time might have influenced the levels of S100B, NF-L, and EVs. Furthermore, although S100B comes primarily from astrocytes, it can be derived from cells outside the nervous system. It is therefore possible that the elevated levels measured in this study originate from cell types other than astrocytes. However, since levels of S100B and astrocyte-derived EVs were significantly positively correlated, this seems unlikely. Moreover, the ELISA assay used in the present study is not sensitive enough to measure levels of S100B in plasma below 0.011 ng/ml. We do not know how the results would have been affected if we had a more sensitive measure of this protein. We did not observe any differences in NF-L levels between the groups, but these NF-L assay results must be interpreted with caution. Although blood sampling and pre-analytical handling were similar in the three groups, and all samples were analyzed simultaneously, ELISA is of limited sensitivity for measuring NF-L in blood^48^. Moreover, a positive control was not available to show whether the assay worked sufficiently beside the standard curve that was in the kit.

The number of participants was relatively small. The study population included fewer men (n = 18) than women (n = 105), which reflects sex differences in the proportion of people diagnosed with stress-related mental disorders^49^. However, the small number of men in the study population meant that it was not possible to determine whether the sex differences we observed, including the raised levels of S100B in women only, were due to chance/sample size or to real sex-related differences.

Another limitation is that we did not adjust for multiple testing, which increases the risk of rejecting a true null hypothesis.

## Conclusion

The results of this study support the findings of previous studies that there might be pathophysiological differences behind SED and MDD and that plasma levels of S100B may be associated with cognitive dysfunction: in the current study, with self-reported symptoms of cognitive failure. We also observed a positive correlation between plasma levels of S100B and plasma levels of astrocyte-derived EVs. This suggests that, like the EVs, elevated levels of S100B derive from astrocytes and may be evidence of pathophysiological changes in these brain cells. Future studies could compare levels of S100B in plasma and in CSF to investigate the potential contribution of blood–brain barrier disruption to stress-related mental disorders. The findings of the current study do not provide evidence that neurons are affected in stress-related disorders, as levels of N-FL were similar in patients with SED, patients with MDD, and healthy controls.

## Supplementary Information

Supplementary Figures.

## Data Availability

The dataset generated and analyzed during the study is available from the corresponding author on reasonable request.

## References

[1] Wang J (2005). Work stress as a risk factor for major depressive episode(s). Psychol. Med..

[2] Soderstrom M, Jeding K, Ekstedt M, Perski A, Akerstedt T (2012). Insufficient sleep predicts clinical burnout. J. Occup. Health Psychol..

[3] Sandstrom A, Rhodin IN, Lundberg M, Olsson T, Nyberg L (2005). Impaired cognitive performance in patients with chronic burnout syndrome. Biol. Psychol..

[4] Kim JJ, Diamond DM (2002). The stressed hippocampus, synaptic plasticity and lost memories. Nat. Rev. Neurosci..

[5] Swedish National Board of Health and Welfare (2022). Swedish version of the International Statistical Classification of Diseases and Related Health Problems, Tenth Revision (ICD-10).

[6] Milenkovic VM, Stanton EH, Nothdurfter C, Rupprecht R, Wetzel CH (2019). The role of chemokines in the pathophysiology of major depressive disorder. Int. J. Mol. Sci..

[7] Blix E, Perski A, Berglund H, Savic I (2013). Long-term occupational stress is associated with regional reductions in brain tissue volumes. PLoS ONE.

[8] Gavelin HM (2020). Mental fatigue in stress-related exhaustion disorder: Structural brain correlates, clinical characteristics and relations with cognitive functioning. Neuroimage Clin..

[9] Murphy-Royal C, Gordon GR, Bains JS (2019). Stress-induced structural and functional modifications of astrocytes-Further implicating glia in the central response to stress. Glia.

[10] Savic I (2015). Structural changes of the brain in relation to occupational stress. Cereb. Cortex.

[11] Mobarrez F, Svenungsson E, Pisetsky DS (2018). Microparticles as autoantigens in systemic lupus erythematosus. Eur. J. Clin. Investig..

[12] Wallensten J (2021). Leakage of astrocyte-derived extracellular vesicles in stress-induced exhaustion disorder: A cross-sectional study. Sci. Rep..

[13] Donato R (2001). S100: a multigenic family of calcium-modulated proteins of the EF-hand type with intracellular and extracellular functional roles. Int. J. Biochem. Cell Biol..

[14] Rezaei O (2017). S100 B: A new concept in neurocritical care. Iran. J. Neurol..

[15] Haselmann V (2021). Plasma-based S100B testing for management of traumatic brain injury in emergency setting. Pract. Lab. Med..

[16] Chmielewska N (2018). Looking for novel, brain-derived, peripheral biomarkers of neurological disorders. Neurol. Neurochir. Pol..

[17] Marchi N (2004). Peripheral markers of blood-brain barrier damage. Clin. Chim. Acta.

[18] Schroeter ML, Abdul-Khaliq H, Diefenbacher A, Blasig IE (2002). S100B is increased in mood disorders and may be reduced by antidepressive treatment. NeuroReport.

[19] Schroeter ML, Abdul-Khaliq H, Krebs M, Diefenbacher A, Blasig IE (2008). Serum markers support disease-specific glial pathology in major depression. J. Affect. Disord..

[20] Arora P (2019). Serum S100B levels in patients with depression. Indian J. Psychiatry.

[21] O'Leary LA, Mechawar N (2021). Implication of cerebral astrocytes in major depression: A review of fine neuroanatomical evidence in humans. Glia.

[22] Bellaver B (2021). Astrocyte biomarkers in Alzheimer disease: A systematic review and meta-analysis. Neurology.

[23] Pham N (2010). Extracranial sources of S100B do not affect serum levels. PLoS ONE.

[24] Undén J (2005). Raised serum S100B levels after acute bone fractures without cerebral injury. J. Trauma.

[25] Khalil M (2018). Neurofilaments as biomarkers in neurological disorders. Nat. Rev. Neurol..

[26] Sandelius Å (2018). Plasma neurofilament light chain concentration in the inherited peripheral neuropathies. Neurology.

[27] Barro C, Chitnis T, Weiner HL (2020). Blood neurofilament light: A critical review of its application to neurologic disease. Ann. Clin. Transl. Neurol.

[28] Puentes F (2017). Neurofilament light as an immune target for pathogenic antibodies. Immunology.

[29] Jakobsson J (2014). Elevated concentrations of neurofilament light chain in the cerebrospinal fluid of bipolar disorder patients. Neuropsychopharmacology.

[30] Spanier S, Kilian HM, Meyer DM, Schlaepfer TE (2019). Treatment resistance in major depression is correlated with increased plasma levels of neurofilament light protein reflecting axonal damage. Med. Hypotheses.

[31] World Health Organization (2019). International Statistical Classification of Diseases and Related Health Problems 10th Revision.

[32] Allgulander, M. W. *et al*. Ågren. Karolinska institutet—Stockholm, Sahlgrenska akademin—Göteborg S. D. L. M.I.N.I. Mini Internationell Neuropsykiatrisk Intervju Svensk version 6.0.0.

[33] Sheehan DV (1998). The Mini-International Neuropsychiatric Interview (M.I.N.I.): The development and validation of a structured diagnostic psychiatric interview for DSM-IV and ICD-10. J. Clin. Psychiatry.

[34] Svanborg P, Asberg M (1994). A new self-rating scale for depression and anxiety states based on the comprehensive psychopathological rating scale. Acta Psychiatr. Scand..

[35] Broadbent DE, Cooper PF, FitzGerald P, Parkes KR (1982). The cognitive failures questionnaire (CFQ) and its correlates. Br. J. Clin. Psychol..

[36] Scaccianoce S, Del Bianco P, Pannitteri G, Passarelli F (2004). Relationship between stress and circulating levels of S100B protein. Brain Res..

[37] Franklin TC, Xu C, Duman RS (2018). Depression and sterile inflammation: Essential role of danger associated molecular patterns. Brain Behav. Immun..

[38] Shi Y, Luan D, Song R, Zhang Z (2020). Value of peripheral neurotrophin levels for the diagnosis of depression and response to treatment: A systematic review and meta-analysis. Eur. Neuropsychopharmacol..

[39] Yang K, Xie GR, Hu YQ, Mao FQ, Su LY (2008). The effects of gender and numbers of depressive episodes on serum S100B levels in patients with major depression. J. Neural Transm. (Vienna).

[40] Savic I, Perski A, Osika W (2018). MRI shows that exhaustion syndrome due to chronic occupational stress is associated with partially reversible cerebral changes. Cereb. Cortex.

[41] Czéh B, Simon M, Schmelting B, Hiemke C, Fuchs E (2006). Astroglial plasticity in the hippocampus is affected by chronic psychosocial stress and concomitant fluoxetine treatment. Neuropsychopharmacology.

[42] Rajewska-Rager A (2021). Longitudinal assessment of S100B serum levels and clinical factors in youth patients with mood disorders. Sci. Rep..

[43] Yu H, Li H, Liu X, Du X, Deng B (2020). Levels of serum S100B are associated with cognitive dysfunction in patients with type 2 diabetes. Aging (Albany).

[44] Barha CK, Hsiung GYR, Liu-Ambrose T (2019). The role of S100B in aerobic training efficacy in older adults with mild vascular cognitive impairment: Secondary analysis of a randomized controlled trial. Neuroscience.

[45] Wang F (2017). orrelation between serum S100β protein levels and cognitive dysfunction in patients with cerebral small vessel disease: A case-control study. Biosci. Rep..

[46] Mobarrez F (2015). CD40L expression in plasma of volunteers following LPS administration: A comparison between assay of CD40L on platelet microvesicles and soluble CD40L. Platelets.

[47] Soop A (2013). Effect of lipopolysaccharide administration on the number, phenotype and content of nuclear molecules in blood microparticles of normal human subjects. Scand. J. Immunol..

[48] Kuhle J (2016). Comparison of three analytical platforms for quantification of the neurofilament light chain in blood samples: ELISA, electrochemiluminescence immunoassay and Simoa. Clin. Chem. Lab. Med..

[49] Rainville JR, Hodes GE (2019). Inflaming sex differences in mood disorders. Neuropsychopharmacology.

