# Supplementary material for: Plasma levels of S100B and neurofilament light chain protein in stress-related mental disorders
Source: Sci Rep. 2022 May 18;12:8339. doi: 10.1038/s41598-022-12287-1 (PMC9117317; doi:10.1038/s41598-022-12287-1)
Supplement: Supplementary file 1 — Supplementary Figures. [file 41598_2022_12287_MOESM1_ESM.pdf]

Supplementary Information

Plasma levels of S100B and neurofilament light chain protein in stress-related mental disorders

Johanna Wallensten, MD; Fariborz Mobarrez, PhD; Marie Åsberg, MD, PhD; Kristian Borg, MD, PhD; Aniella Beser, MSc; Alexander Wilczek MD, PhD; Anna Nager, MD, PhD

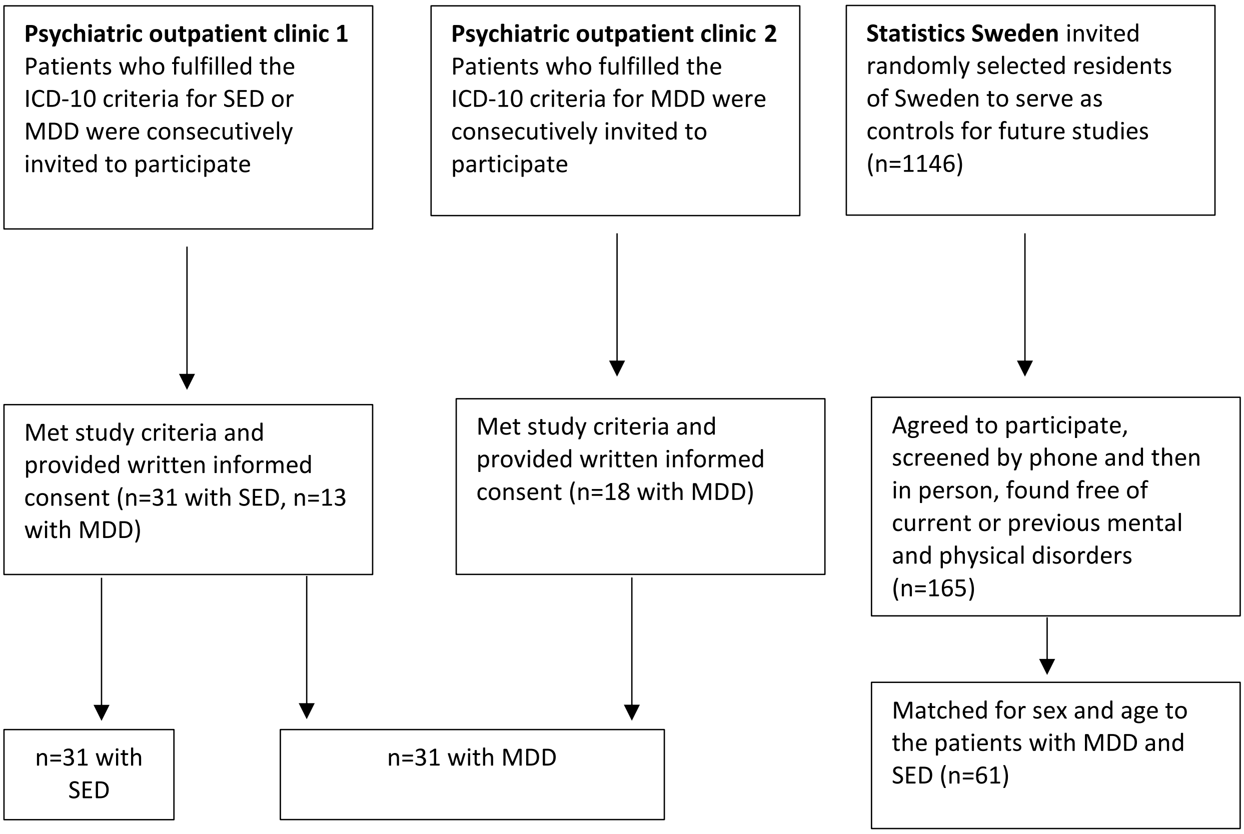

Supplementary figure 1. Study design. Initially, the study recruited patients from Psychiatric outpatient clinic 1. To increase the number of patients with MDD in the study, patients from Psychiatric outpatient clinic 2 were also invited to participate.

ICD-10, Swedish version of the International Classification of Diseases, tenth version; SED, stress-induced exhaustion disorder; MDD, major depressive disorder.

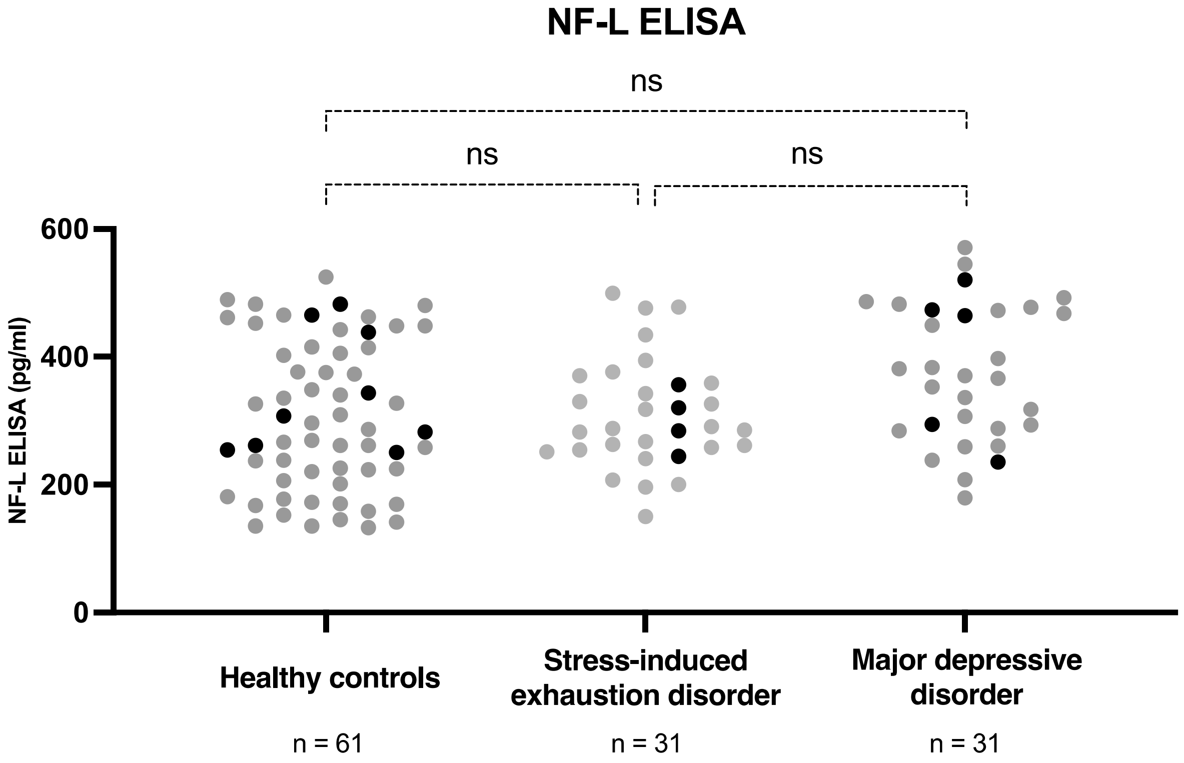

Supplementary figure 2. Plasma levels of neurofilament light chain protein (NF-L) measured by enzyme-linked immunosorbent assay (ELISA). Gray dots represent women and black dots represent men.
